# Barry-Perkins-Young syndrome

**DOI:** 10.11604/pamj.2024.47.191.42932

**Published:** 2024-04-16

**Authors:** Ashwin Karnan, Anjana Ledwani

**Affiliations:** 1 Department of Respiratory Medicine, Jawaharlal Nehru Medical College, Datta Meghe Institute of Higher Education and Research, Sawangi (Meghe), Wardha, Maharashtra, India

**Keywords:** Cough, infertility, sinusitis, bronchiectasis

## Image in medicine

A 28-year-old male presented with complaints of fever, cough with expectoration and difficulty in breathing for the past 5 days. Patient is a fruit seller by occupation and gives history of similar illness in the past due to recurrent sinusitis and a significant treatment history for infertility for the past 2 years. Computed tomography of the chest showed tree in bud appearance predominantly in the upper lobes, with cystic and tractional bronchiectasis changes in the bilateral lower lobes. A transrectal ultrasound confirmed obstructive azoospermia for which he was advised vasoepididymostomy. A diagnosis of Young´s syndrome was made, and he was treated with intravenous antibiotics. Young´s syndrome also known as Barry-Perkins-Young syndrome or sinusitis-infertility syndrome is a rare, inherited syndrome commonly seen in middle-aged men with chronic rhinosinusitis, nasal polyps, infertility and bronchiectasis. It is named after Dr. Donald Young, a urologist who first described it. The exact cause is unknown, but childhood exposure to mercury and genetic factors are speculated. Treatment involves control of sinus, lung infection and surgical/assisted measures for reproduction.

**Figure 1:**
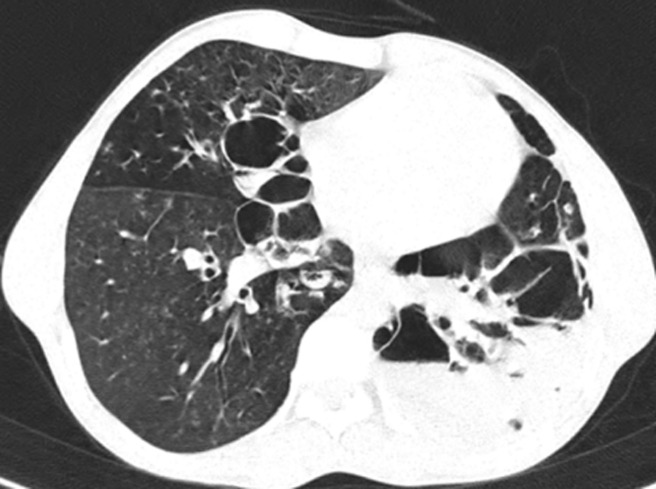
computed tomography of the chest showing bilateral variable-sized cystic and tractional bronchiectatic changes

